
JOURNAL INFORMATION
==============================
NLM Title Abbreviation: Halls J Health
NLM Journal ID: 0011644

Hall's Journal of Health

Publisher: Biomedical Journal Digitization Project

ARTICLE INFORMATION
==============================
PMCID: PMC9235757
PMID: 36491761
Article version: 1
Subjects: Articles

Whooping Cough Cured

Print publication date: 1886 Apr
Volume: 33
Issue: 4
First page: 128
Last page: 128
License: This work is free of known copyright restrictions. For more information, please see PMC Back Issue Digitization.
License URL: https://creativecommons.org/publicdomain/mark/1.0/

==============================

Whooping Cough Cured.

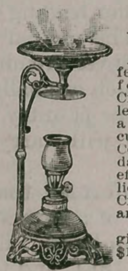

PAGE'S VAPORIZER.
A simple yet perfect apparatus for Vaporizing Cresolene. Cresolene vaporized in a closed room cures WHOOPING COUGH in a few days. It is also effectual in relieving Asthma, Croup, Diphtheria and Scarlet Fever.
Ask your druggist for it. Price, $1.50.

W. H. Schieffelin & Co., Sole Agents, 170 William St., New York.
